# Assessment of Oral Hygiene in Patients Using Fixed and Removable Dentures Treated at the University Dental Clinic in Krakow

**DOI:** 10.3390/ijerph182211986

**Published:** 2021-11-15

**Authors:** Joanna Ryniewicz, Magdalena Orczykowska, Krzysztof Gronkiewicz, Małgorzata Pihut

**Affiliations:** 1 Department of Integrated Dentistry, Institute of Dentistry, Faculty of Medicine, Jagiellonian University Medical College, 31-155 Kraków, Poland; 2 Department of Prosthodontics, Institute of Dentistry, Faculty of Medicine, Jagiellonian University Medical College, 31-155 Kraków, Poland; magdalena.orczykowska@uj.edu.pl (M.O.); krzysztof.gronkiewicz@uj.edu.pl (K.G.); malgorzata.pihut@uj.edu.pl (M.P.)

**Keywords:** oral hygiene, dentures, dental plaque, removable denture hygiene, fixed denture hygiene, denture cleaning agents

## Abstract

Apart from local lesions, the presence of dental plaque may also have an effect on health, especially in people with general diseases. The aim of this project was to assess the oral hygiene of patients prosthetically treated at the Dental Prosthetics Clinic of the University Dental Clinic in Krakow (Poland) using fixed and removable dentures and to determine the demographic relationships and data related to the education of the respondents. The research material consisted of 120 patients who used fixed (group I) and removable (group II) restorations. Basic dental examinations and oral hygiene examinations were carried out with the use of the API (Approximal Plaque Index) and PI (Plaque Index) plaque indices. This study presents the percentage of respondents in terms of sex, place of residence, and education. The mean PI (Plaque Index) values were 46.73% (Group I) and 50.05% (Group II). (*p* = 0.4839). The mean values of API (Approximal Plaque Index) amounted to 65.14% (Group I) and 68.94% (Group II) (*p* = 0.4695). Patients using dentures showed insufficient oral hygiene, and the hygiene status of patients did not depend on the type of dentures used. The group that is most often treated with prosthetics is women with secondary education. The most numerous group of patients at the Dental Prosthetics Clinic are people living in large cities which results from easier access to health care.

## 1. Introduction

The specificity of the prosthetic treatment of patients with missing teeth is based on the use of fixed and removable prosthetic restorations. These dentures enable the patient to chew and speak and improve their facial aesthetics. They also have an impact on the patient’s emotional sphere [1]. However, dentures operating in the stomatognathic system may, if patients do not maintain proper hygiene, have a negative impact on the local condition of the oral cavity and the patient’s general health. Only a few hours after inserting the prosthesis into the oral cavity, the accumulation of bacterial plaque on its surface and in the area of the dentition was observed [2]. Within the bacterial biofilm, streptococci are dominant, alongside staphylococci (both Gram-positive and Gram-negative strains) and Candida albicans fungi [3]. The excessive growth of microorganisms leads to a lowering of the saliva pH and a reduction in the protective properties of the oral mucosa [4,5].

Bacterial plaque may have a negative effect on the local condition of the oral cavity, causing inflammation and cavities or caries of the abutments. These changes may also affect soft tissues, appearing in the form of gingivitis and periodontal disease, and, with the coexistence of other etiological factors, they favor the induction of prosthetic stomatopathy in the mucosa [5]. The unfavorable influence of the plaque bacterial flora may also be important in patients using dental implants, leading to the development of periimplantitis. The microbes of the bacterial and fungal plaque also contribute to the occurrence of oral mycosis and may lead to the formation of halitosis [6,7,8,9,10].

The mechanism of action of plaque bacteria on hard tooth tissues is related to the decrease in pH below 5.5. This is the result of the production of acids, including lactic acid, formic acid, and acetic acid, by the bacteria. This condition causes enamel demineralization and starts the caries process. With time, bacteria and their metabolic products penetrate deep into the tooth, initiating the process of disintegration of the organic part of hard tissues and the further demineralization of their inorganic substance [8]. The mechanism of action of bacteria in the area of the soft tissues of the oral cavity is of a general nature, beyond the known local significance. An example of this is the influence of plaque bacteria on the development of gingivitis and periodontitis, where the host’s immune and inflammatory response is induced [9]. Immune reactions are important not only in defense mechanisms, but also in the destructive effect they have on the patient’s own tissues. The cascade of host responses also affects the general health of the patient, especially in sensitive individuals and those taking immunosuppressive drugs. The influence of periodontal diseases on heart and circulatory system diseases, problems during pregnancy, cerebral hemorrhage, respiratory system infections, and diabetes has been proven previously [2,7,10,11,12,13,14,15,16,17].

In order to reduce the risk of the above-mentioned diseases, it is necessary to accurately determine the condition of patients’ oral cavity hygiene and prostheses and learn about their hygiene habits. Appropriate tools for monitoring the state of hygiene are hygiene indices, which are convenient tool for determining the amount of plaque both in the area of the teeth and in the area of dentures [3,5,11,18,19,20,21,22,23].

The aim of this project was to assess the status of the oral hygiene of patients treated prosthetically at the Dental Prosthetics Clinic of the University Dental Clinic in Krakow (Poland) using fixed and removable dentures and to determine the demographic relationships and data related to the education of the respondents. The null hypothesis H0 was applied: that there is a statistically significant difference between the distribution of the two groups—users of fixed and removable dentures. In the alternative hypothesis H1, there is no statistically significant difference between the distribution of the groups.

## 2. Materials and Methods

The study material consisted of 120 patients treated at the Dental Prosthetics Clinic who were generally healthy (without aggravating diseases), who were aged 40 to 57 years, who used fixed dentures (group I: 30 women, 14 men), and who used removable restorations (group II: 50 women, 26 men). The patients used removable acrylic dentures and fixed metal dentures veneered with ceramics. The sample size was calculated with the following assumptions: the α significance level was assumed to be 0.05 and the test power was assumed to be 0.8.

The inclusion criteria were: patients who were the appropriate age, who used fixed and removable dentures, who had good general health, and who gave their consent to participate in the research project. The exclusion criteria were: patients who had general diseases that prevented them from continuing to participate in the research, who did not want to participate in the research, who showed the loss of subsequent teeth, and who had an indication for the use of full dentures. Allocation to the study groups took place sequentially, in accordance with the inclusion criteria, during the qualification of patients for prosthetic treatment.

The following examinations were conducted: basic dental examination and oral hygiene study with the use of an in-house questionnaire, in which the oral hygiene assessment was based on the use of the interdental plaque index according to the Lange API (Approximal Plaque Index) and the bacterial plaque index PI (Plaque Index) [24,25]. In the API study, the presence of plaque in the interdental spaces was assessed, then what percentage of all examined spaces was occupied by the plaque was calculated. The presence of the plaque was visualized either on an explorer or after the staining of the blooms with staining tablets or a solution. After all the spaces were assessed, the number of spaces with the plaque was divided by the number of all spaces tested and multiplied by 100 to obtain the percentage. The internal interdental spaces (palatal/lingual) in the 1st and 3rd quarters and the outer surfaces (labial/buccal) in the 2nd and 4th quarters were assessed. Each space received an appropriate score (0–1), then the space values were summed and divided by the number of all tested spaces; the result was given as a percentage. Only places that formed contact points were examined.

**Figure 1:**
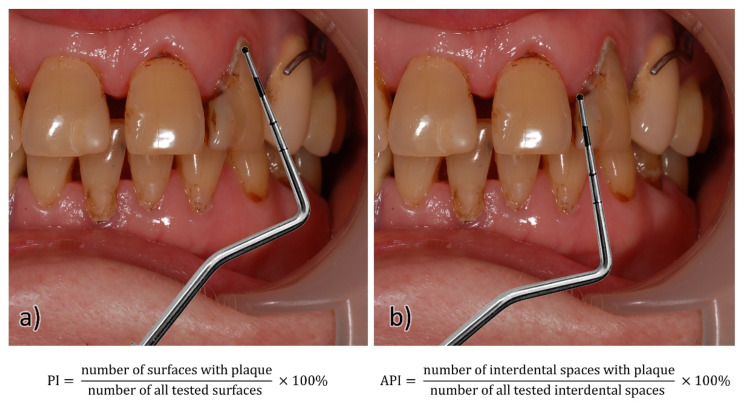
Scheme used for assessing the presence of plaque on the (**a**) smooth surface of the tooth (PI) and (**b**) in the interdental space (API). Equations show indices calculations.

On the other hand, the PI indicator was used to assess the presence of plaque in the area of the tooth neck, from where it is most difficult to remove. The presence of plaque was assessed on the buccal/labial and lingual/palatal surfaces and on two contact surfaces (proximal and distal). The presence of the plaque on the four above-mentioned surfaces was assessed with a score (0–1). Then, the number of surfaces with the plaque was added up and divided by the total number of surfaces tested. The result was expressed as a percentage outcome, as shown in Figure 1.

The evaluation of the survey in our study was carried out with the use of a specialized package of PQStat computer programs, taking into account the division of the respondents into two groups and based on classic calculation procedures: mean values and median scores, standard deviations, and minimum and maximum values. To conduct the analyses, the following criteria were established: the homogeneity of variance and the normality of distribution. The analysis of variance test and the Tukey’s post hoc test for dependent variables were carried out as measures of the significance level (the null hypothesis H0: there is a difference between the groups; the alternative hypothesis H1: there is no difference between the groups). The level of significance was set to 0.05. Pearson’s chi-square test and comparisons between groups were used to carry out the demographic analysis.

All procedures carried out in the study involving human participants were performed in accordance with the guidelines of the Declaration of Helsinki.

Consent of the Bioethics Committee to conduct research: 1072.6120.117.2021, dated 16 June 2021.

## 3. Results

### 3.1. The Results of the Demographic Analysis

The age range of the studied patients ranged from 40 to 57 years. However, the average age of the respondents was 49.04 years for both groups. Group I consisted of 44 people, and group II consisted of 76 people. In group I, there were 30 women, who made up 68% of the group, and there were 14 men, who made up 32%. In group II, there were 50 women, who made up 66% of the respondents, and 26 men, who made up 34% of the group (no statistically significant differences between the groups, *p* = 0.864). Among the patients in group I, most had a secondary education—46% (20 respondents). This was followed by those with higher education, at 31% (14 respondents), and then those with primary education, at 23% (10 respondents). The majority of group II also had secondary education—52% (40 respondents). People with primary and higher education accounted for 24% each (18 respondents) (no statistically significant differences between the groups, *p* = 0.649). The majority of group I lived in large cities—66% (29 respondents), while inhabitants of rural areas accounted for 25% (11 respondents). The smallest number of patients in this group lived in small towns—9% (4 respondents). A similar quantitative distribution in terms of the place of residence could be observed in group II. The largest number were people living in large cities—58% (44 respondents); after this were patients living in the countryside, at 25% (19 respondents), while the smallest number were patients living in small towns, at 17% (13 respondents) (no statistically significant differences between the groups, *p* = 0.501).

Among the women, most were patients with secondary education, at 61% (49 respondents), followed by patients with higher education, at 26% (21 respondents). The smallest number were those with primary education, at 13% (10 respondents). Among the men, the highest percentage were patients with primary education, at 45% (18 respondents), while people with higher education and secondary education accounted for 27.5% each (11 respondents) (the number of women with secondary education was statistically significant in relation to the total number of prosthetically treated people, *p* < 0.001).

The largest number of prosthetically treated people lived in a large city, at 61% (73 respondents), while much smaller shares lived in rural areas (25%, 30 respondents) and small towns (14%, 17 respondents).

### 3.2. Oral Hygiene Assessment Results

**Figure 2:**
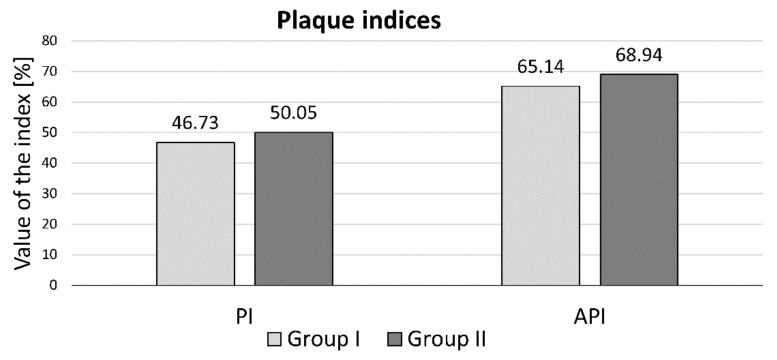
The mean values of the plaque indices in groups I and II.

**Figure 3:**
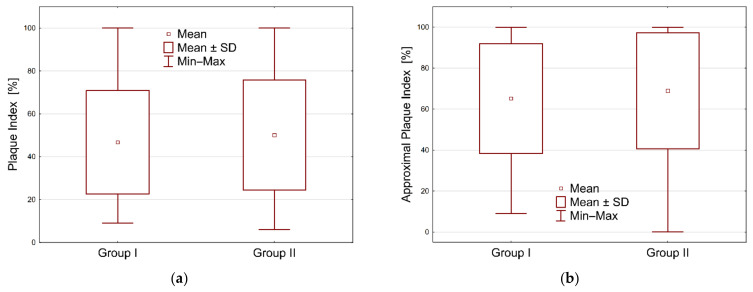
Graphical representation of indices through boxplots in two groups: (**a**) plaque index, (**b**) approximal plaque index.

The values of the PI index ranged from 9 to 100 in group I and from 6 to 100 in group II. In the case of the API index, the values ranged from 9 to 100 in group I and from 0 to 100 in group II. The mean values of the bacterial plaque index (PI) in group I was 46.73%, with a standard deviation of 24.15. In group II, the mean value was 50.05%, with a standard deviation of 25.62. The above results do not differ statistically significantly—*p* = 0.4839. The mean value of the approximal plaque index (API) in group I was 65.14% with a standard deviation of 26.83, while in group II it was 68.94% with a standard deviation of 28.29. In the case of the API index, the results between the groups did not differ statistically significantly—the *p* value was 0.4695. For both the analyzed PI and API indices, hypothesis H0 was rejected while the alternative hypothesis H1 was accepted: there is no statistically significant difference between the two groups of users of fixed and removable dentures. See Table 1 and Figure 2 and Figure 3.

## 4. Discussion

The demographic analysis of the studied groups was different for other authors. Taraszkiewicz-Sulik noted a similar percentage of women and men in his study (women 51%, men 49%) as opposed to Szalewski, whose gender distribution was 68.2% for women and 31.8% for men [18]. Szalewski’s data [19] was consistent with our own research. With regard to the level of education, Taraszkiewicz-Szulik found that the largest share was respondents with secondary and post-secondary education (38.3%), followed by 37% of respondents with higher education and 24.6% of respondents with primary and vocational education, which was consistent with the results of our own research. Different results were observed by Szelowski, who determined the following percentage distribution in the group of patients using removable dentures: 56.4% of the respondents had primary and vocational education, 37% of the respondents had secondary education, and 6.6% of the respondents had higher education. The number of respondents analyzed according to the place of residence was different in the research of other authors than in our own research. Taraszkiewicz-Sulik determined that, among the respondents, the largest number were inhabitants of small towns, at 46.6%, while 35.4% of the respondents lived in large cities and 17.8% were rural residents. Szalewski drew attention to the similar percentages of respondents living in large cities, rural areas, and small and medium-sized towns—at 36.4%, 33.3%, and 30.3%, respectively. These data differed from the data found in our own research, where the largest percentages were inhabitants of large cities, at 66% in group I and 58% in group II [18].

This study could be limited by the quantitative differences between the groups. However, a statistical comparative analysis of both groups in terms of sex, place of residence, and education did not show statistically significant differences.

The average age of society, including prosthetic patients, is continuously increasing, and thus there are indications for the use of fixed and removable restorations in patients in cases where implantoprostheses cannot be used. This is often associated with professional activity and the awareness of the need to replace lost teeth, as well as an increase in the material status of patients [2,7,26]. Fixed metal-ceramic and removable dentures are the most commonly used restorations despite the significant development of the technology used for manufacturing implant prostheses and non-metal ceramics. A very important element of prosthetic treatment is the appropriate construction of prostheses to enable the maintenance of proper hygiene, as well as the information provided to patients regarding the principles, stages, and importance of adhering to and maintaining proper oral hygiene and prostheses. The dates of follow-up visits, during which the doctor should check the patient’s hygiene status, also play a significant role [1,5,8,13]. Improper hygiene causes the development of inflammations of the oral mucosa, periodontopathy, the development of prosthetic stomatopathy, and caries of other teeth. This is often combined with an unpleasant odor from the mouth (fetor ex ore). Moreover, the use of plate dentures additionally creates favorable conditions for the growth of bacteria and fungi under the plate of the prosthesis due to the increased temperature, as well as the lowered saliva pH, the porosity of acrylic material, and the consumption of significant amounts of carbohydrates. Additionally, preparations for the maintenance of dentures may constitute a reservoir of bacteria and fungi (colonization of microorganisms) if dentures are not properly cleaned [2,8,13,15].

Pathogenic microorganisms can be associated with the occurrence of numerous general diseases, such as endocarditis, myocardial infarction, pneumonia, aspiration pneumonia, peptic ulcer disease, reflux, and osteoporosis. Additionally, immunosuppression, antibiotic therapy, and radiotherapy have negative effects. It has been proven that cytokines and interleukins released from denture plaque can be transported to the lungs, which contributes to the development of inflammation in the respiratory system [15]. Hygienic negligence is a significant source of infection of the lower respiratory system by the aspiration of microorganisms from the denture plaque and nasopharyngeal mucosa. On the other hand, Andrea et al. proved the presence of a significant number of microorganisms responsible for the development of obstructive pulmonary disease in the denture plaque and teeth. Moreover, oral cavity infections can induce a chronic state of insulin resistance [10]. The presence of Streptococus sanguis and Porphyromonas gingivalis bacteria may be responsible for platelet aggregation and thus cause blood clots and atherosclerosis. This, in turn, increases the level of C-reactive protein CRP, which increases the risk of a heart attack [2,11,16].

The data from the literature show that regardless of patients’ level of education and type of profession, they do not know the basic rules of dental and oral hygiene. Additionally, iatrogenic errors in prosthetic treatment (incorrect design of prostheses, incorrect material) can aggravate prosthetic negligence. An important element that negatively affects the condition of the oral cavity is malnutrition in geriatric patients, in whom hygienic negligence leads to a significant deterioration of the general condition [3,7,18,19,20].

The results of the hygiene status concerning the API index turned out to be particularly disturbing in our own research. This level approached the value of 70% (65.14 in group I; 68.94 in group II), at which oral hygiene is clearly defined as bad. With regard to the value of the API index, the results shown in the literature are at a similar level. Czerniawska-Kliman et al., in a study on a group of 365 patients, obtained a comparable mean API value of 63% [12]. A similar result for the mean API value was also obtained by Olszewska-Czyż et al. In their group of 100 patients, the value was 63.07% [14]. Głowacka et al., who conducted a study in a group of 332 patients aged 65–74 years, achieved an average API score of 67.6%, which is the most consistent value with their own research [15].

The PI value in our own work, which was at a level of 46.73 in group I and 50.05 in group II, was characterized by patient’s insufficient level of hygiene, in which nearly 50% of the tooth surface was not properly cleaned. In the case of the PI index, similar mean values of the index were observed in Głowacka et al. (at the level of 51.9% and lower) and in Kim et al. (38%) in the age group of 58 and 59 years. In the group of young patients, this value turned out to be similar to our own research, reaching the value of 56% [15].

In conclusion, regarding the unsatisfactory oral hygiene of prosthetic patients, it is necessary to point out the need for continuous education in the field of oral hygiene and its importance for general health and the prevention of the development of inflammatory and oncological lesions in the oral cavity, as well as to increase the control of hygiene during routine prosthetic visits, which is essential for the general health and local condition of patients [27,28,29,30].

## 5. Conclusions

Patients using dentures show insufficient levels of oral hygiene, and the unfavorable condition of the oral hygiene of patients does not depend on the type of prosthesis used. The group that is most often treated with prosthetics is women with secondary education. The majority of patients at the Dental Prosthetics Clinic are people living in large cities due to their easier access to health care.

## Figures and Tables

**Table 1:** Plaque index values gained from the assessment of statistical significance obtained in groups I (patients using fixed dentures) and II (patients using removable restorations).

Range of Plaque Indices							
	Group I (*n* = 44)			Group II (*n* = 76)			
	Mean ± SD	Min–Max	Median	Mean ± SD	Min–Max	Median	*p*
PI	46.73 ± 24.15	9–100	41.50	50.05 ± 25.62	6–100	50.00	0.4839
API	65.14 ± 26.83	9–100	62.50	68.94 ± 28.29	0–100	70.50	0.4695

PI: plaque index; API: approximal plaque index; SD: standard deviation.

## Data Availability

Data available on request from the corresponding author due to restrictions (privacy and ethical).
